# Deconstructing subjective unmet healthcare needs: a South Korean case study with policy implications

**DOI:** 10.3389/fpubh.2024.1385951

**Published:** 2024-05-10

**Authors:** Woojin Chung

**Affiliations:** ^1^Department of Health Policy and Management, Graduate School of Public Health, Yonsei University, Seoul, Republic of Korea; ^2^Korea Peace Institute, Seoul, Republic of Korea

**Keywords:** unmet healthcare needs, universal health coverage, sample selection model, Korea Health Panel, South Korea

## Abstract

**Background:**

Despite widespread efforts by many countries to reduce the prevalence of unmet healthcare needs within their populations, there remains a scarcity of research systematically exploring the components of these needs.

**Objectives:**

This study aims to deconstruct subjective unmet healthcare needs into two distinct components: the experience of subjective healthcare needs (the “Needs” component) and the experience of unmet needs contingent on those healthcare needs (the “Unmet” component).

**Methods:**

This analysis utilizes data from 13,359 adults aged 19 or older, collected through the 2018 Korea Health Panel survey, with the aim of minimizing the influence of the coronavirus disease 19 pandemic. The two dependent variables are the experience of subjective healthcare needs and whether these needs have been met. The independent variables include 15 socio-demographic, health, and functional characteristics. The study employs both a population proportion analysis and a multivariable bivariate probit model with sample selection.

**Results:**

In South Korea, 11.6% (CI [confidence interval] = 11.0–12.3%) of the population experienced subjective unmet healthcare needs. Upon deconstructing these, 96.7% (CI = 96.2–97.1%) of the population exhibited the Needs component, and 12.0% (CI = 11.4–12.7%) displayed the Unmet component. Each independent variable showed different associations between the two components. Furthermore, effective interventions targeting the characteristics associated with each component could reduce the proportion of the population experiencing subjective unmet healthcare needs from 11.6 to 4.0%.

**Conclusion:**

South Korea faces a significant challenge due to the considerable prevalence of subjective unmet healthcare needs. To address this challenge effectively, the universal healthcare coverage system should adapt its approach based on the characteristics associated with both the Needs and Unmet components of subjective unmet healthcare needs. To achieve this goal, it is highly recommended that the government prioritize strengthening community-based primary healthcare, which currently suffers from insufficient resources.

## 1 Introduction

Universal health coverage (UHC) continues to be a crucial objective in contemporary global health policy. The UHC system aims to provide essential, high-quality healthcare and ensures unrestricted access to safe, effective, and affordable medications and vaccinations, regardless of an individual’s socio-demographic status (1, 2). This system is anticipated to improve access to healthcare services for those previously unable to do so, thereby reducing overall unmet healthcare needs in the population (3, 4).

The measurement of unmet healthcare needs in a population is often conducted from the perspective of subjective unmet healthcare needs (SUN, hereafter). In this context, SUN is derived from survey responses collected from a diverse cross-section of the population, assessing whether they perceived a need for healthcare in the past 12 months and, subsequently, whether they were unable to access it. The primary rationale for the widespread utilization of SUN data is their ability to gather information on unmet healthcare needs from individuals, regardless of their status as patients or non-patients (5–9).

Despite significant efforts by most countries to address unmet healthcare needs within their populations, data from the Organization for Economic Co-operation and Development (OECD) reveals a wide variation in the prevalence of SUN across countries. For instance, as of 2019, Spain reported a mere 0.2 percent SUN rate. In contrast, Greece and Estonia registered 8.5 percent and 15.5 percent, respectively (5). Numerous studies conducted in diverse countries have examined the individual characteristics associated with SUN, focusing on gender, age, marital status, education, income, economic activity, health insurance, self-rated health, chronic illnesses, depression, and functional limitations (6–10).

However, prior research has not extensively explored the two key components of SUN: the experience of subjective healthcare needs (the “Needs” component, hereafter) and the experience of unmet needs contingent on those healthcare needs (the “Unmet” component, hereafter). While one study delved into the role of social capital in addressing SUN across the entire population of 20 European countries (11), a more nuanced understanding of the associations between different characteristics and SUN continues to elude us, especially at the country-specific level. To effectively reduce SUN within a country’s population, governments must undertake comprehensive efforts to address either the Needs component, the Unmet component, or both, considering the intricate associations between these components and individuals’ characteristics. Therefore, this study aims to bridge the gap between previous research and the requirements for policies to reduce SUN across various countries. This study utilized a nationally representative survey dataset focused on healthcare utilization in South Korea (Korea, hereafter) to achieve this goal. Additionally, it employs a multivariable bivariate probit model with sample selection to analyze the study sample. Furthermore, based on the resulting findings, this study compares the proportions of population individuals experiencing SUN, the Needs component, and the Unmet component between European countries and Korea.

The anticipated outcomes of this study include guiding researchers in formulating and evaluating novel theories related to healthcare access by dissecting SUN into its Needs and Unmet components. Additionally, these findings are expected to provide policymakers in each country with insights to assist their populations in reducing SUN compared to that in the previous initiatives. This study is part of a research series aimed at contributing to the development of each country’s UHC systems by addressing the challenges posed by rapid demographic and social changes (7, 8, 12).

## 2 Overview of South Korea’s universal health coverage system

Korea introduced its social health insurance program following a Bismarck-type model with multiple payers, similar to the systems used in Germany, France, and Japan. The National Health Insurance (NHI) system, established in 1989, plays a pivotal role in achieving UHC. Complementing this, the Medical Care Aid (MCA) program, designed to assist the economically disadvantaged, covers approximately 3% of the population. In 2000, a transition occurred, consolidating the NHI into a single-payer system, and the NHI and the MCA are now overseen by the National Health Insurance Service (NHIS). This centralization of governance ensures uniformity in contribution and benefit packages nationwide (7, 13).

In Korea, approximately 90% of medical facilities are privately owned, and physicians and hospitals are primarily reimbursed through a fee-for-service payment model. The government refrained from regulating the number of hospital beds nationwide (13). These factors contribute to high rates of healthcare utilization. In 2019, the annual doctor consultations *per capita* stood at 17.2, the highest among 34 OECD member countries (average 6.8). The average length of hospital stays was 18.0 days, which was the highest among 38 OECD member countries (with an average of 7.6 days). Additionally, the consumption of second-line antibiotics (measured in terms of the defined daily dose per 1,000 people) reached 9.4 in 2019, second only to Greece’s 10.6 among 30 OECD member countries (with an average of 3.3) (5).

While Korea’s UHC system excels in objective measures of physical health status, it falls short of assessing citizens’ emotional and mental well-being. In 2019, life expectancy at birth reached 83.3 years, surpassing the OECD member countries (with an average of 81.0 years). However, 15.2% of adults rated their health as “bad” or “very bad,” the second-highest among 36 OECD member countries (with an average of 8.5%). Similarly, the suicide rate per 100,000 people (age-standardized) was 24.6, the highest among the 38 OECD member countries (with an average of 11.0) (5).

In 2019, despite the population aged 65 and above accounting for 14.9%, which is lower than the OECD member-country average of 17.3%, Korea witnessed the most rapid growth in this age group among OECD member countries. Consequently, the annual growth rate in health expenditure *per capita* between 2015 and 2019 reached 7.8%, ranking second highest among the 38 OECD member countries (with an average of 2.7%). As a result, health expenditure as a percentage of the gross domestic product reached 8.2% in 2019, closely approaching the OECD member-country average of 8.8% (5).

Primary healthcare (PHC) effectively addresses various healthcare needs, provides non-emergency care, acts as a gatekeeper for secondary or tertiary care, prevents avoidable hospital admissions, reduces unnecessary healthcare utilization and expenditure, and improves outcomes (14). However, in stark contrast to most OECD countries, Korea’s UHC system lacks a well-established framework for PHC (13, 15). In 2019, the proportion of general practitioners among all medical doctors was merely 6%, the lowest among 32 OECD member countries (with an average of 23%) and approximately one-eighth of Canada’s 47%. Consequently, most physicians in Korea are specialists, although their numbers are limited. The density of practicing doctors per 1,000 people was 2.5, which is significantly below the OECD member-country average of 3.6 (5). Consequently, individuals seeking healthcare typically first encounter specialists across various healthcare facilities authorized to provide outpatient and inpatient services competitively (13, 16).

## 3 Methods

### 3.1 Data

Data for this study were extracted from the Korea Health Panel (KHP) Survey (version 1.7), a nationally representative survey encompassing the non-institutionalized civilian population. The KHP survey, jointly administered by the NHIS and the Korea Institute of Health and Social Affairs (a government research institution), operates under the supervision of the Ministry of Health and Welfare. Since 2008, the survey has been conducted biennially. The survey selects households via a two-stage cluster probability sample method guided by population census data provided by the National Statistical Office. The survey collected information from all eligible household members on the following: individual healthcare utilization, health-related expenses, socio-demographic characteristics, lifestyle choices, and health-related attributes. Data were collected through personal computer-aided interviews conducted annually on designated weekdays.

In this study, data from 2018 was utilized to avoid potential transient effects introduced by the emergence of the coronavirus disease 19 (COVID-19) pandemic in 2019. The initial dataset included 14,262 observations of individuals aged over 19 years. However, 903 observations lacking information on the SUN were excluded, resulting in a final study sample of 13,359 observations, representing 93.7% of the original dataset.

To conduct a comparative analysis between Korea and European countries regarding SUN, the Needs component, and the Unmet component, this study assessed data from 22 European countries using the European Union Statistics on Income and Living Conditions (EU-SILC) dataset (9). These data encompassed individuals aged 16 years and older, and data for 2018 were analyzed to mitigate the potentially varying influence of the COVID-19 pandemic across diverse countries. Among these 22 European countries, 18 were ultimately selected for comparative analysis, with the Czech Republic, Slovenia, Spain, and Germany excluded because of variations in the SUN experience questionnaire used in the EU-SILC survey.

This study used secondary data obtained from the KHP survey, which is publicly available on the survey website (https://www.khp.re.kr:444/eng/main.do; accessed on May 20, 2020). All the interviewees in the survey were anonymous, and all procedures involving human participants followed the ethical standards of the relevant institutional and national research committee and the 1964 Declaration of Helsinki and its later amendments, or comparable ethical standards.

### 3.2 Variables

#### 3.2.1 Dependent variables

In the KHP survey questionnaire, a specific question was asked: “Have you ever experienced not receiving the necessary medical treatment or examination (excluding dental care) in the past year (12 months)?” Respondents had several answer options: Option (1): “Yes, I have experienced it at least once.” Option (2): “No, I have not experienced it” and Option (3): “No medical treatment or examination of any kind was needed.”

Two dependent variables were created based on the responses. First, for the Needs component, the individuals were categorized into two groups. Those who chose Option (1) or Option (2) were classified as the “Needs” group, while those who selected Option (3) were placed in the “non-Needs” group. Subsequently, a binary dependent variable was established, with a value of “1” for individuals in the “Needs” group and “0” for those in the “non-Needs” group.

Second, regarding the Unmet component, a second dependent variable was formed among individuals in the “Needs” group. Those who answered Option (1) had healthcare needs; however, they did not receive the necessary care, indicating unmet healthcare needs. These individuals constituted the “Unmet” group. Conversely, those who selected Option (2) experienced healthcare needs and received the necessary care and were categorized as the “non-Unmet” group. A binary dependent variable was then created for individuals in the “Needs” group, with a value of “1” for individuals in the “Unmet” group and “0” for those in the “non-Unmet” group.

#### 3.2.2 Independent variables

This study employed a comprehensive set of independent variables, including socio-demographic, health, and functional characteristics, largely consistent with previous research (7, 8, 12), with minor adjustments tailored to the specific objectives of this study.

##### 3.2.2.1 Sociodemographic characteristics

- Gender: Man or woman
- Age: 19–44 years, 45–64 years, 65 years or older
- Marital Status: Married or unmarried (including never married, separated, widowed, or divorced)
- Residential Region: Northern (Seoul, Incheon, Gyeonggi-do, Gangwon-do), central (Daejeon, Sejong, Chungcheongbuk-do, Chungcheongnam-do), western (Gwangju, Jeollabuk-do, Jeollanam-do, Jeju-do), or eastern (Busan, Daegu, Ulsan, Gyeongsangbuk-do, Gyeongsangnam-do)
- Education: Less than college or college and higher
- Occupation: No job (unemployed or not engaged in economic activity), blue-collar job, or white-collar job
- Household Income: Lowest quintile, medium (three middle quintiles), or highest quintile (adjusted for household size using the square root equivalence scale)
- National Health Security Program Status: NHI or MCA
- Private Health Insurance Holding Status: Yes or no

##### 3.2.2.2 Health and functional characteristics

- Current Smoking: Yes or no
- Alcohol Consumption: Yes or no
- Regular Physical Exercise: Yes or no (defined as participating in any of the three types of physical exercises—walking, moderate-level, or high-level exercise—for 30 min or longer, at least three times a week)
- Obesity: Yes or no (defined as a body mass index (BMI) of at least 25 kg/m^2^ according to the Asia-Pacific criteria for obesity status provided by the World Health Organization Western Pacific Region) (17).
- Self-Assessed Health Status: Poor or no (among the options of “excellent, very good, fair, poor, or very poor”)
- Presence of Chronic Disease: Yes or no (based on self-reported responses regarding physician diagnosis)
- Functional Limitations: Yes or no (based on an individual’s response to the question, “Is your daily living routine (conducting work, housekeeping, study, and social, leisure, or familiar activities) limited due to a disease or an injury?”)

### 3.3 Analytical strategy

Acknowledging that experiencing healthcare needs is a prerequisite for SUN is essential. Without this prerequisite, the model could be affected by a sample selection bias, potentially leading to inefficient estimates (18). To address potential sample selection bias, this study initially employed the Heckman two-step estimation method, which is commonly used for such purposes. However, Heckman’s estimator is unsuitable due to sensitivity to multicollinearity between the estimated Mill’s ratio and independent variables (19), and its applicability is further complicated by a binary dependent variable in the second step of the procedure.

Therefore, this study used a multivariable bivariate probit model with sample selection consisting of two sequential equations (20). The first equation, associated with the Needs component, deals with a binary dependent variable that determines whether an individual has experienced healthcare needs. In contrast, the second equation, associated with the Unmet component, concerns another binary dependent variable that determines whether the healthcare needs of the individuals who experienced them were unmet. This two-equation system addresses both unconditional and conditional situations. The unconditional situation determines whether an individual experiences healthcare needs based on one set of independent variables and one error term, whereas the conditional situation assesses whether the healthcare needs of the individuals who experience them remain unmet, relying on another set of independent variables and another error term. The error terms were assumed to follow a standard bivariate normal distribution with a correlation coefficient. Estimates of the coefficients associated with the independent variables and correlation coefficients were obtained using the full-information maximum likelihood estimation method.

After establishing the statistical model, the study addressed several statistical aspects. Regular physical exercise was excluded from the second equation to account for identification restrictions in the multivariable bivariate probit model with sample selection (21, 22). Population proportions and their 95% confidence intervals (CIs) for individuals experiencing SUN, the Needs component, and the Unmet component were estimated (23). Variance inflation factor values were reduced to 2.15 in both the first and second equations to mitigate multicollinearity (24). The best-fitting model was selected through comparison with Akaike information criterion (25). Using the average marginal effects analysis method (26), predictive probabilities and their 95% CIs were estimated, representing the probabilities of individuals experiencing SUN, the Needs component, and the Unmet component. Lastly, the study compared the proportions of individuals experiencing SUN, the Needs component, and the Unmet component between Korea and 18 European countries. For Korea, a population proportion analysis was conducted to make these comparisons. Unlike European countries, Korea did not provide necessary data for direct comparisons with the proportions of the 18 European countries (9).

Cross-sectional weights from KHP survey data were used in the analysis. Statistical significance was defined as *p* < 0.05, and analyses were conducted using the SAS software (version 9.4; SAS Institute, Cary, NC, United States) and STATA 17 software (StataCorp, College Station, TX, United States).

## 4 Results

### 4.1 Summary statistics and population proportions of experiencing SUN, the Needs component, and the Unmet component

In the initial statistical analysis, the categories showing the highest proportion in each variable are: women, individuals aged 45–64 years, married individuals, residents in the northern region, individuals with education levels below college, those with blue-collar jobs, individuals with a medium level of household income, people covered by the NHI, individuals holding a private health insurance plan, non-smokers, individuals with no regular physical exercise, those who are not obese, individuals with no poor self-assessed health, those without chronic diseases, and those with no functional limitations (columns 1 and 2 in Table 1).

**Table 1:** Summary statistics and population proportions of experiencing healthcare needs and experiencing unmet needs contingent on healthcare needs.

Characteristic	All individuals			Unmet needs contingent on healthcare needs (%)
	*n*	(%)	Healthcare needs (%)	
All			96.7	12.0
Gender			<0.001	0.218
Woman	7,217	(54.0)	98.7	13.2
Man	6,142	(46.0)	96.2	11.5
Age (years)			<0.001	<0.001
19–44	4,240	(31.7)	94.7	10.5
45–64	4,972	(37.2)	98.2	12.5
65 and over	4,147	(31.1)	99.8	14.2
Marital status			<0.001	0.689
Married	8,797	(65.9)	98.7	11.8
Unmarried	4,562	(34.1)	95.5	13.7
Residential region			<0.001	<0.001
Northern	5,505	(41.2)	96.6	14.2
Central	1,686	(12.6)	97.8	11.6
Western	2,183	(16.3)	98.1	11.3
Eastern	3,985	(29.8)	98.5	10.9
Education			0.008	0.047
Lower than college	8,935	(66.9)	98.0	13.0
College or higher	4,424	(33.1)	96.7	11.3
Occupation			0.141	0.017
No job	5,219	(39.1)	97.8	12.5
Blue-collar job	5,478	(41.0)	97.7	12.7
White-collar job	2,662	(19.9)	96.8	11.6
Household income			0.028	<0.001
Lowest quintile	2,677	(20.0)	98.8	17.5
Medium	8,021	(60.0)	97.4	11.3
Highest quintile	2,661	(20.0)	96.7	10.6
National health security program			0.030	<0.001
National Health Insurance	12,920	(96.7)	97.5	11.9
Medical Care Aid	439	(3.3)	98.4	26.8
Private health insurance			0.004	0.071
No	3,415	(25.6)	97.5	14.3
Yes	9,944	(74.4)	97.6	11.8
Current smoker			<0.001	<0.001
No	11,083	(83.0)	98.1	12.0
Yes	2,276	(17.0)	95.1	14.6
Alcohol consumer			<0.001	0.995
No	4,601	(34.4)	98.6	13.6
Yes	8,758	(65.6)	97.0	11.8
Regular physical exercise			0.501	0.673
No	8,647	(64.7)	97.7	12.9
Yes	4,712	(35.3)	97.3	11.4
Obese			0.253	0.022
No	9,729	(72.8)	97.6	12.1
Yes	3,630	(27.2)	97.4	13.3
Poor self-assessed health			<0.001	<0.001
No	11,322	(84.8)	97.2	10.5
Yes	2037	(15.2)	99.8	22.4
Chronic disease			<0.001	<0.001
No	4,946	(37.0)	94.4	10.1
Yes	8,413	(63.0)	99.4	13.7
Functional limitation			<0.001	<0.001
No	12,509	(93.6)	97.4	11.1
Yes	850	(6.4)	99.6	30.9

*N* = 13,359 individuals. All population proportions were estimated using a complex sampling design. For brevity, 95% confidence intervals of population proportions are not included. *p*-values were calculated using Pearson Chi-square test.

The proportion of people experiencing SUN was estimated to be 11.6% (CI = 11.0–12.3%). The proportion of the Needs component was 96.7% (CI = 96.2–97.1%). This proportion varied significantly across the categories for all characteristics except occupation, regular physical exercise, and obesity (columns 3 and 4 in Table 1). Furthermore, the proportion of the Unmet component was 12.0% (CI = 11.4–12.7%). This proportion varied significantly across the categories for all characteristics except marital status, private health insurance, alcohol consumption, and regular physical exercise (columns 5 and 6 in Table 1).

### 4.2 Proportions of people experiencing SUN, the Needs component, and the Unmet component in selected countries

**Figure 1:**
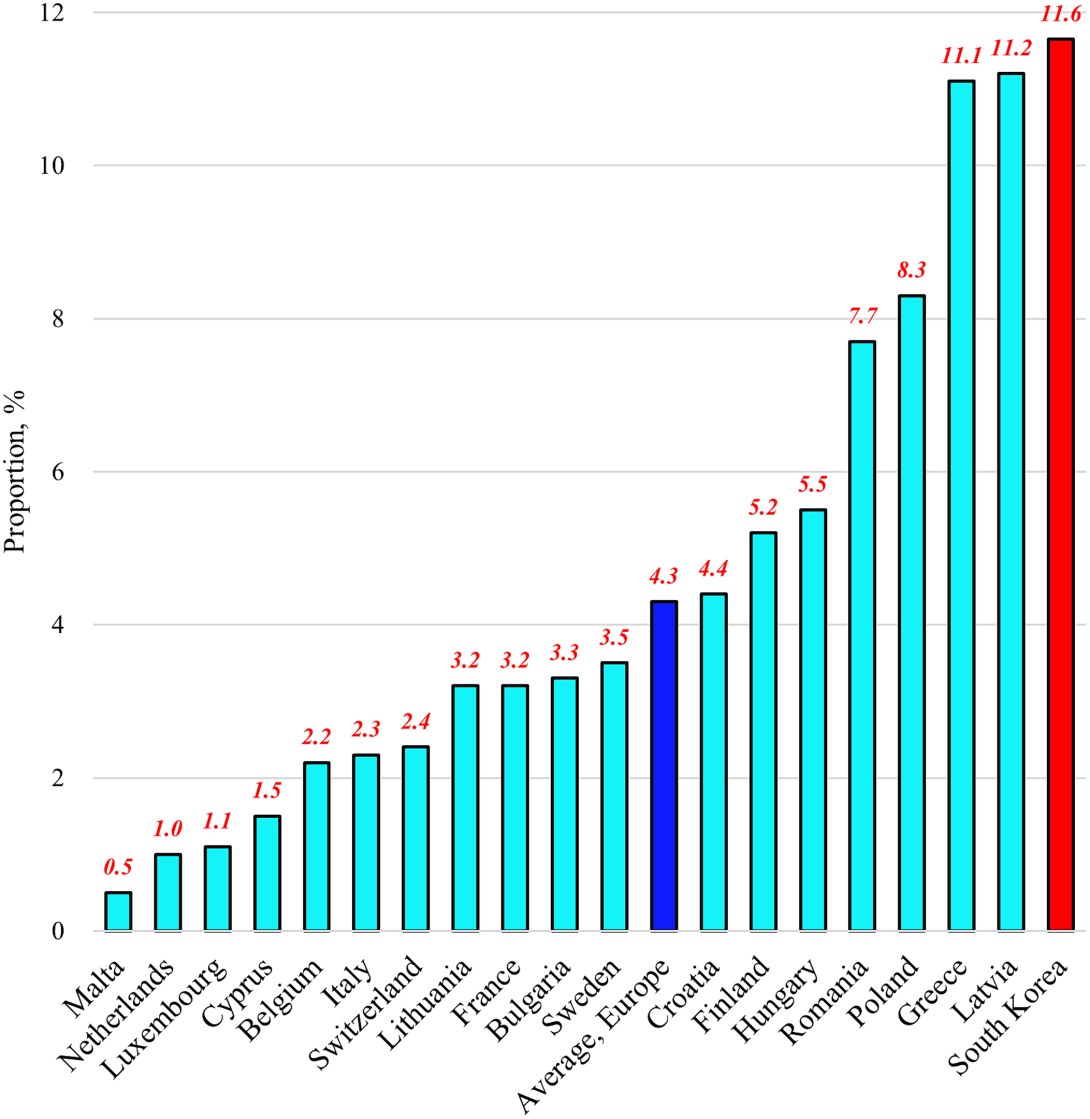
Proportions of people experiencing “unmet healthcare needs” in selected countries as of 2018.

**Figure 2:**
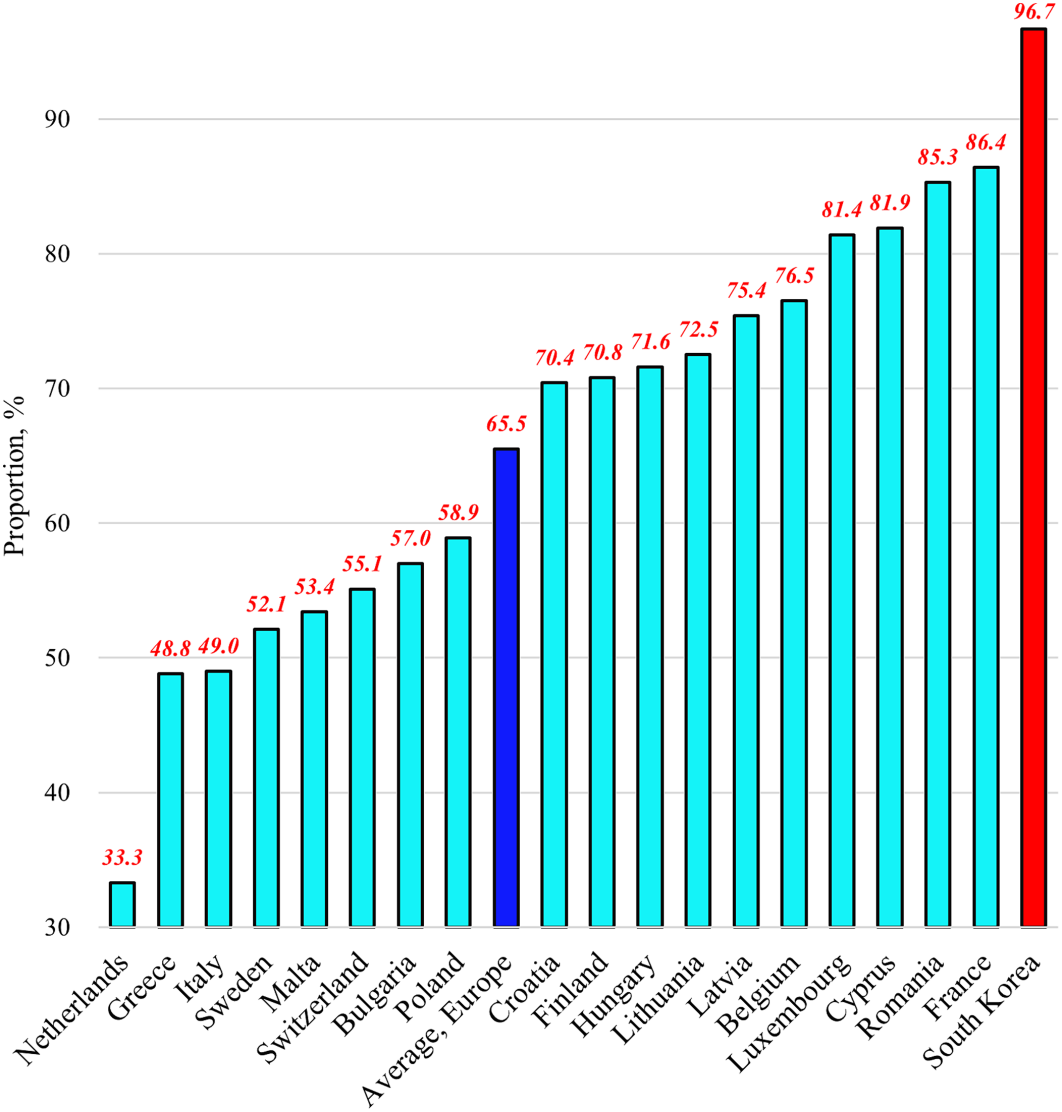
Proportions of people experiencing “healthcare needs” in selected countries as of 2018.

**Figure 3:**
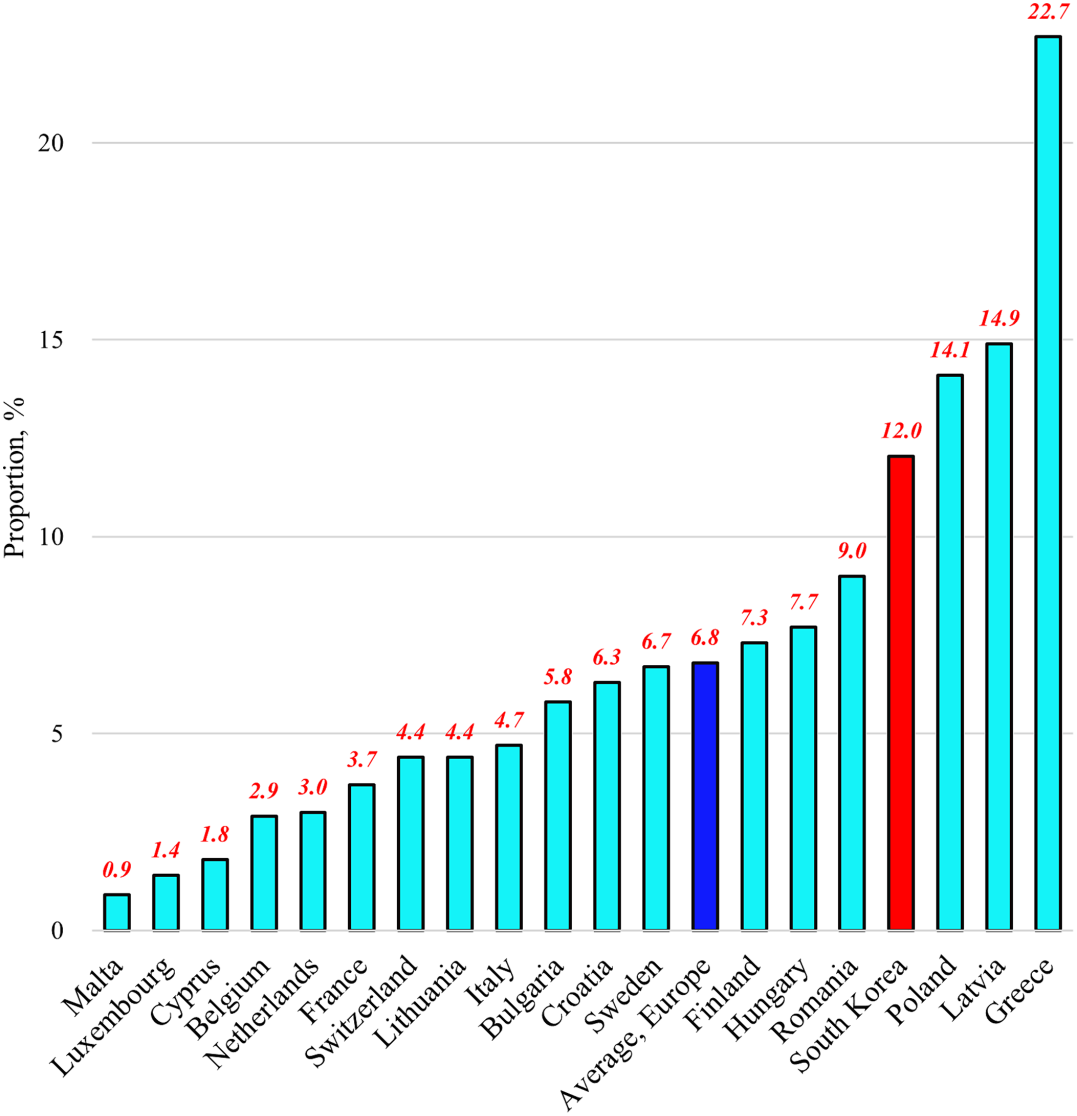
Proportions of people experiencing “unmet needs contingent on healthcare needs” in selected countries as of 2018.

Among the 18 European countries analyzed in this study, the average proportion of people experiencing SUN in 2018 was 4.3% (Figure 1). This proportion ranges from 0.5% in Malta to 11.2% in Latvia. By contrast, Korea had a proportion of 11.6%. For the proportion of the Needs component, the 18 European countries had an average value of 65.5% (Figure 2). This proportion ranges from 33.3% in the Netherlands to 86.4% in France. By contrast, Korea had a proportion of 96.7%. Regarding the proportion of the Unmet component, the 18 European countries had an average of 6.8% (Figure 3). This proportion ranges from 0.9% in Malta to 22.7% in Greece. Korea’s proportion was 12.0%.

### 4.3 Characteristics associated with the Needs component

Various individual characteristics were associated with the Needs component (columns 1 through 3 in Table 2). Men exhibited a lower likelihood of the Needs component compared to that in women (coefficient [COEF], −0.360; *p* < 0.001). The likelihood was higher for individuals aged 45–64 (COEF, 0.122; *p* = 0.024) and those aged 65 years and older (COEF, 0.641; *p* < 0.001) than that for individuals aged 19–44. Unmarried individuals had a decreased likelihood compared to that in married individuals (COEF, −0.415; *p* < 0.001). Living in the central (COEF, 0.169; *p* < 0.001), western (COEF, 0.362; *p* < 0.001), and eastern regions (COEF, 0.299; *p* < 0.001) represented an increased likelihood compared to living in the northern region. Holding a private health insurance plan was associated with an elevated likelihood (COEF, 0.251; *p* < 0.001). Current smokers had a lower likelihood compared to current non-smokers (COEF, −0.162; *p* < 0.001). Conversely, individuals with poor self-assessed health had a higher likelihood than those without (COEF, 0.688; *p* < 0.001). Individuals with a chronic disease had a lower likelihood compared to those without it (COEF, −0.630; *p* < 0.001). However, having a functional limitation increased the likelihood compared with that in not having functional limitations (COEF, 0.356; *p* = 0.003).

**Table 2:** Associations of characteristics with healthcare needs and unmet needs contingent on healthcare needs.

Characteristic	Healthcare needs			Unmet needs contingent on healthcare needs		
	COEF	(SE)	*p*-value	COEF	(SE)	*p*-value
Man (REF: Woman)	−0.360	(0.019)	<0.001	−0.055	(0.030)	0.062
Age (REF: 19–44 years)						
45–64	0.122	(0.054)	0.024	−0.018	(0.045)	0.687
65 and over	0.641	(0.029)	<0.001	−0.076	(0.077)	0.327
Unmarried (REF: Married)	−0.415	(0.043)	<0.001	0.061	(0.019)	0.001
Residential region (REF: Northern)						
Central	0.169	(0.013)	<0.001	−0.183	(0.013)	<0.001
Western	0.362	(0.008)	<0.001	−0.257	(0.009)	<0.001
Eastern	0.299	(0.004)	<0.001	−0.235	(0.005)	<0.001
College or higher (REF: Lower than college)	0.097	(0.085)	0.254	0.021	(0.028)	0.457
Occupation (REF: No job)						
Blue-collar job	0.100	(0.080)	0.215	0.220	(0.039)	<0.001
White-collar job	0.037	(0.064)	0.565	0.153	(0.045)	0.001
Household income (REF: Medium)						
Lowest quintile	−0.059	(0.059)	0.323	0.160	(0.076)	0.036
Highest quintile	−0.044	(0.088)	0.614	−0.074	(0.077)	0.336
MCA (REF: NHI)	0.207	(0.227)	0.364	0.276	(0.129)	0.032
Private health insurance, yes (REF: No)	0.251	(0.068)	<0.001	0.020	(0.033)	0.536
Current smoker, yes (REF: No)	−0.162	(0.037)	<0.001	0.224	(0.043)	<0.001
Alcohol consumer, yes (REF: No)	0.050	0.060	0.401	0.079	(0.039)	0.043
Regular physical exercise, yes (REF: No)	0.021	(0.055)	0.697	–	–	–
Obese, yes (R: No)	−0.074	(0.056)	0.191	0.065	(0.035)	0.063
Poor self-assessed health, yes (REF: No)	0.688	(0.070)	<0.001	0.362	(0.057)	<0.001
Chronic diseases, no (REF: Yes)	−0.630	(0.012)	<0.001	0.098	(0.017)	<0.001
Functional limitation, yes (REF: No)	0.356	(0.120)	0.003	0.501	(0.110)	<0.001
Constant	2.160	(0.077)	<0.001	−1.357	(0.056)	<0.001
Correlation coefficient	−0.880	(0.017)	<0.001			

*N* = 13,359 individuals. All values were estimated using a complex sampling design. *p*-values were obtained by Pearson Chi-square test.COEF, estimates of coefficients; SE, robust estimates of standard errors; REF, reference category; MCA, Medical Care Aid; NHI, National Health Insurance.

### 4.4 Characteristics associated with the Unmet component

Regarding the likelihood of the Unmet component (columns 4 through 6 in Table 2), unmarried individuals demonstrated a higher likelihood compared to that in married individuals (COEF, 0.061; *p* < 0.001). Living in the central region (COEF, −0.183; *p* < 0.001), western region (COEF, −0.257; *p* < 0.001), or eastern region (COEF, −0.235; *p* < 0.001) represented a reduced likelihood compared to that in the northern region. Having a blue-collar job increased the likelihood compared to that in being jobless (COEF, 0.220; *p* < 0.001), while having a white-collar job decreased the likelihood (COEF, −0.153; *p* = 0.001). Those in the lowest quintile of household income were more likely to exhibit the Unmet component compared to those in the medium-income group (COEF, 0.160; *p* = 0.036). MCA beneficiaries were more likely to do so compared to NHI beneficiaries (COEF, 0.276; *p* = 0.032). Current smokers (COEF, 0.224; *p* < 0.001) and alcohol consumers (COEF, 0.079; *p* = 0.043) encountered an increased likelihood compared to their contrary counterparts. Individuals with poor self-assessed health had a higher likelihood than those without (COEF, 0.362; *p* < 0.001). Individuals with chronic diseases had a higher likelihood than those without (COEF, 0.098; *p* < 0.001). However, having functional limitations increased the likelihood compared to that in not having functional limitations (COEF, 0.501; *p* < 0.001).

## 5 Discussion

### 5.1 Insights from international comparisons of proportions of people experiencing SUN, the Needs component, and the Unmet component

The results of this study, covering 18 European countries and Korea, reveal that Korea had higher proportions of people experiencing SUN (11.6%) and the Needs component (96.7%) compared to that in any other European country. In addition, the proportion of the Unmet component in Korea (12.0%) exceeded the European average (Figures 1–3). These findings provide valuable insights into the unique challenges faced by the Korean UHC system and offer important lessons for addressing healthcare access issues. To reduce SUN in the Korean population, the government should consider a comprehensive reform of the country’s UHC system, focusing on both healthcare needs and the unmet needs contingent on those healthcare needs. However, it is particularly important to prioritize efforts to reduce healthcare needs, as they represent a more significant portion of the population.

### 5.2 Four groups of characteristics associated with the Needs component and/or the Unmet component

For each characteristic, it is important to emphasize its association with the Needs component may or may not differ from its association with the Unmet component. Therefore, this study categorizes their associations into four distinct groups: Group A comprises characteristics associated with the Needs component but not the Unmet component; Group B comprises characteristics associated with the Unmet component but not the Needs component; Group C comprises characteristics associated with both the Needs and Unmet components; and Group D comprises characteristics associated with neither the Needs component nor the Unmet component, such as education level and obesity.

Group A included characteristics such as gender, age, and private health insurance status. Women were more likely than men to exhibit the Needs component, consistent with previous studies (27, 28). Several reasons may contribute to this finding: women tend to have higher morbidity rates, are more likely to rate their health as poor or very poor, are more health conscious, and are more proactive in seeking healthcare for their conditions (28, 29). Older individuals appear to require more healthcare services, possibly because of increased health concerns associated with aging and declining health (11). However, a recent study in Korea showed a negative relationship between age and healthcare needs in men aged 20–35 (8). This could be attributed to intense job demands and competition in Korea’s labor market, which may lead younger men to neglect their healthcare needs (8, 30). Holding a private health insurance plan may increase healthcare needs. This could be because individuals who purchase private health insurance may be more health-conscious or because having private health insurance potentially leads to moral hazards in healthcare utilization (31).

Characteristics such as occupation, household income, national health security program status, and alcohol consumption fell into Group B. Among individuals experiencing healthcare needs, those in the labor market (either in blue- or white-collar jobs) were more likely to experience unmet needs than those who did not. This finding aligns with previous research showing that employed individuals use healthcare services less frequently than unemployed individuals because of the limited time available for healthcare visits, which can be influenced by living conditions or workplace environments (8, 32).

The likelihood of their healthcare needs going unmet was higher for individuals in the lowest quintile of household income than for those in the medium-income group. Similarly, it was higher for MCA program recipients than for NHI beneficiaries. Individuals with a lower socioeconomic status tend to face challenges in accessing healthcare when needed, often due to the financial burden of healthcare expenses (10, 33–36). The healthcare needs of alcohol consumers were more likely to be unmet than those of nonconsumers. Due to time constraints during outpatient visits, a few individuals may turn to alcohol consumption as a coping mechanism for health issues, even when they recognize the need for healthcare (37).

Group C included characteristics such as marital status, residential region, current smoking status, self-assessed health, chronic disease, and functional limitations. Within Group C, both poor self-assessed health and having a functional limitation exhibit consistent associations with the Needs and Unmet components. Individuals reporting poor health or functional limitations are more likely than their counterparts to experience both the Needs and Unmet components. This may be because these individuals are more likely to have low incomes, making it difficult to afford healthcare expenses (10, 33–36). Inadequate treatment due to financial constraints can lead to an ongoing vicious cycle in which individuals continue to experience healthcare needs without meeting them, further deteriorating their health.

In contrast, marital status, residential region, current smoking, and chronic disease show different associations between the Needs and Unmet components. Unmarried individuals, compared to that in married individuals, show a lower likelihood of the Needs component; however, they had a higher likelihood of the Unmet component. This result aligns with previous findings suggesting that marriage may increase healthcare needs through spousal care and encouragement to visit a physician (38, 39). Residents in the three other regions were more prone to demonstrate the Needs component than those in the northern region; however, they showed a lower likelihood of displaying the Unmet component. In particular, the disparity between the northern and western regions was striking. These findings warrant further investigation as they may be related to regional disparities in healthcare systems such as stewardship, financing, resource allocation, and service delivery (12, 40). Current smokers may be less likely to seek healthcare for health issues than current non-smokers (never or ex-smokers) (41). This may explain why current smokers are less likely to be associated with the Needs component and more likely to be associated with the Unmet component. Similarly, individuals without chronic diseases may be less likely to seek healthcare for health issues than those with chronic diseases. This may explain why individuals without chronic diseases are less likely to exhibit the Needs component and more likely to display the Unmet component.

Based on these results, this study examined how the Korean government could reduce SUN by intervening in specific individual characteristics. To address this question, the study identified categories of characteristics that can reduce SUN by lowering either the Needs component, the Unmet component, or both based on the results from the multivariable analysis (Table 2). Additionally, it estimated the predictive probabilities (along with their 95% CIs) for experiencing SUN when individuals belonged to each of the selected categories. Finally, Wald tests were used to assess whether the predictive probabilities of experiencing SUN were significantly lower than the current level of 11.6%.

Consequently, the following characteristic categories have the potential to significantly decrease the predictive probability of experiencing SUN from the current level of 11.6%.

- Lowering only the Needs component: Being a man (to 10.4%) and not having private health insurance (to 10.3%)
- Lowering only the Unmet component: Having no job (to 8.9%) and being a non-alcohol consumer (to 10.3%)
- Raising the Needs component and lowering the Unmet component: Living in the western region (to 9.6%) and being a current non-smoker (to 10.9%)
- Lowering both the Needs and Unmet components: Having non-poor self-assessed health (to 10.4%), and having no functional limitation (to 11.0%)

Considering all the mentioned categories of characteristics in Korea (excluding gender, which is not influenced by policies), the predictive probability of experiencing SUN decreased from the current level of 11.6 to 4.0%.

### 5.3 Addressing SUN reduction: establishing a foundation for community-based primary healthcare in Korea

Based on the previously mentioned categories of characteristics, this study developed specific strategies for reducing unmet healthcare needs in Korea as follows:

- Discourage individuals from obtaining private health insurance plans to reduce the Needs component.
- Provide assistance to jobless individuals to consult a doctor to reduce the Unmet component.
- Improve health promotion efforts to encourage alcohol consumers to reduce or quit alcohol consumption to lower the Unmet component.
- Detect healthcare needs and help coordinate treatments for people living in other regions using the methods used for people living in the western region to increase the Needs component and lower the Unmet component.
- Improve health promotion efforts to encourage cigarette smokers to reduce or quit smoking to increase the Needs component and lower the Unmet component.
- Work on reducing the number of individuals who perceive their health as poor even when they are healthy to reduce both the Needs and Unmet components.
- Implement preventive measures to reduce the incidence of functional limitations to lower both the Needs and Unmet components.

To implement these strategies, Korea must establish a foundation for community-based PHC. Strengthening PHC is essential for universal healthcare access because it encompasses various healthcare dimensions and is a fundamental element of health systems (1, 2, 42, 43). The transformation of healthcare in Korea requires addressing five pivotal issues: persuasion and promotion, resource allocation, organizational changes, incentives, and measurement tools (44).

First, a critical aspect is transitioning from a disease-centric healthcare model to one centered on people. The government must garner support from stakeholders and the public to emphasize the advantages of expanding PHC, highlighting the importance of generalism, inclusivity, and continuity of care. Strategies should be devised to navigate potential opposition to this transformative process (42, 43, 45). Second, policies should prioritize increasing the total number of doctors and ensuring the equitable distribution of PHC doctors to ensure an adequate workforce. Leveraging nurse practitioners and pharmacists trained in PHC for initial patient interactions is essential, particularly in underserved areas (5, 46, 47).

Third, the adoption of team-based PHC delivery models is highly recommended. Multidisciplinary PHC teams comprising doctors, nurses, and pharmacists effectively address complex healthcare needs. Digital technology should be harnessed to facilitate proactive treatment and enhance access to care (44, 48, 49). Fourth, establishing payment mechanisms that promote quality PHC and incentivize providers to deliver people-centered care is crucial. Exploring various models based on the local healthcare environment, such as fee-for-service, pay-for-performance, bundled payments, and population-based payments, is essential (44, 50). Fifth, developing comprehensive measurement tools (including patient experience measurements) is essential to improving the quality of PHC. Collaboration with other developed countries is indispensable for advancing research methodologies related to PHC (47, 51–54). By addressing these pivotal aspects, Korea can lay a robust foundation for community-based PHC and effectively contribute to SUN reduction.

### 5.4 Limitations

This study has certain limitations. Firstly, self-reported SUN data may be subject to recall bias (10, 23, 55). Secondly, respondents who lacked information on SUN were excluded from the study. This exclusion reduced the proportion of individuals aged <45 years from 34.5 to 31.7% in the final study sample. The age distributions were significantly different before and after the exclusion (*p* = 0.002). Therefore, the results of this study should be interpreted with this consideration. In contrast, gender distribution did not differ significantly. Thirdly, the scope of the study was restricted by the lack of information regarding the assurance of unmet healthcare needs by the public health security system, the specific symptoms associated with SUN, and demographic factors such as race, immigration status, religious affiliation, religious devotion, social capital, social support, and emotional satisfaction with healthcare (11, 33, 56).

Fourthly, to maintain simplicity and clarity in achieving the objectives of this study, gender- or age-specific analyses were not conducted. Fifthly, the Korean data included individuals aged 19 years or older. In contrast, the European national data included individuals aged 16 years or older. This age discrepancy should be considered when interpreting the results of this study. Sixthly, comparisons with Japan were impossible due to the unavailability of Japanese data. Japan shares many demographic, social, and cultural characteristics with Korea and exhibited a similar unmet medical need experience rate in 2018. Future research should further explore this topic. Finally, data from 2018 was analyzed to minimize the impact of the COVID-19 pandemic. Subsequent studies using more recent data should examine the validity of these findings.

## 6 Conclusion

This study describes a comprehensive analysis of subjective unmet healthcare needs in Korea, marking the first utilization of Korean data to dissect these needs into two essential components: the experience of subjective healthcare needs and the experience of unmet needs contingent on those needs. In addition, it offers a valuable comparison of these experiences with those of European countries. These findings underscore the significance of targeted public health strategies to address the characteristics associated with each of these two components, thereby reducing the prevalence of subjective unmet healthcare needs in the Korean population. These strategies are closely linked to strengthening community-based primary healthcare, which is currently lacking in the Korean universal healthcare coverage system. The insights gained from this study are expected to provide policymakers in each country with valuable information regarding their healthcare coverage systems and enable them to establish more nuanced, targeted public health policies to promote equitable healthcare access for their populations.

## Data availability statement

Publicly available datasets were analyzed in this study. Data is from the Korea Health Panel survey, which is available to the scientific community with a signed data access agreement from the Korea Institute for Health and Social Affairs and the National Health Insurance Service database (https://www.khp.re.kr:444/eng/main.do, accessed on 20 May 2020).

## Ethics statement

The studies involving humans were approved by the Institutional Review Board of the Severance Hospital, Seoul, Republic of Korea. The studies were conducted in accordance with the local legislation and institutional requirements. Written informed consent for participation was not required from the participants or the participants’ legal guardians/next of kin in accordance with the national legislation and institutional requirements.

## Author contributions

WC: Conceptualization, Data curation, Formal analysis, Funding acquisition, Investigation, Methodology, Project administration, Resources, Software, Supervision, Validation, Visualization, Writing – original draft, Writing – review & editing.
